# Mothers' experiences of giving medicines to children with severe and profound intellectual disabilities—The impact on time

**DOI:** 10.1111/cch.12960

**Published:** 2022-01-25

**Authors:** Carmel Doyle

**Affiliations:** ^1^ School of Nursing & Midwifery Trinity College Dublin Dublin Ireland

**Keywords:** child, intellectual disability, medication administration, mothers

## Abstract

**Background:**

Chronic health conditions experienced by children with severe and profound intellectual disabilities are accompanied by numerous challenges because of the prolonged period over which children take medication and the large number of drugs they take. Mothers experience many challenges in giving medicines, from difficulties in physical administration to manipulation of medication, covert administration and alternative formulations. The aim of this study was to explore mothers' lived experience of giving medicines to children with severe and profound intellectual disabilities.

**Methods:**

A hermeneutic phenomenological approach was used. Semistructured face‐to‐face interviews and participant diaries were adopted for data collection, resulting in 28 interviews undertaken and 7 diaries completed with mothers of children with severe and profound intellectual disabilities. Van Manen's method for thematic analysis was used for data analysis.

**Results:**

The concept of time and the impact of giving medicines were apparent, mothers being ‘always on call’ and the constant full‐time pace of their caring role evident. There was little spontaneity in their lives, dampened by the routine of giving medicines and their caregiving role. The necessity to be prepared and organized was highlighted as important in ensuring children got their medications on time and safely.

**Conclusions:**

This study provides insight into the phenomenon of mothers' lived experience of giving medicines to children with severe and profound intellectual disabilities. It has enabled exploration and familiarity with the lifeworld of mothers and offers meaning on the phenomena of giving medicines. It was concluded that this experience is a relentless and challenging one, yet appears invisible as an element of care in professional discourse. Through addressing the gap in understanding and exploring the meaning of this phenomenon, it may be useful in developing care for mothers and children with severe and profound intellectual disabilities.

**Key messages**

- Health professionals need to develop an understanding of the task of giving medicines and the associated time required to manage these medicines on a daily basis, appreciating the challenges experienced by mothers.
- Health professionals such as the general practitioner, pharmacist and hospital are key in providing supportive elements to mothers' daily role.
- Mothers need to be able to get a break from the task of giving medicines achievable through adequate in‐home and out‐of‐home respite care.
- Formal education and training need to be developed to encompass all elements related to giving medicines, in turn empowering mothers to be confident and competent in this task.

## 1 INTRODUCTION

The rate of newborns and children with intellectual disabilities who have life‐limiting conditions and complex physical healthcare needs has increased significantly (D'Amore et al., 2011; Health Service Executive [HSE], 2018; Milligan, 2010), with approximately 1106 children with a severe or profound intellectual disability in Ireland (Hourigan et al., 2018). This growth is a result of increased life expectancy, ongoing developments in healthcare knowledge, drugs, advanced medical care, technology, parental efforts and availability of varied facilities for education and living (Brenner et al., 2018, 2020; HSE, 2009; McCarron et al., 2011; McConkey et al., 2007; Nakken & Vlaskamp, 2007; Nicholl et al., 2013; Simkiss, 2011). Many of these children have rare syndromes and multiple and complex needs and require high levels of assistance and support (Eddy, 2013; Gates & Mafuba, 2014). Consequently, many children are becoming increasingly dependent on equipment or technological devices to sustain their life or optimize health involving a myriad of equipment, technology and medications, managed by parents at home (Brenner et al., 2018, 2020; Doyle, 2020a; Toly et al., 2012).

Chronic health conditions experienced by children with severe and profound intellectual disabilities are accompanied by numerous challenges because of the prolonged period over which children take medication and sometimes the large number of drugs they take (Kalyango et al., 2012). Children with complex needs receive 5 times the number of medications than typical children (Fiks et al., 2012), and this can include a variety of medications: protein pump inhibitors for gastro‐oesophageal reflux, prokinetic agents for dysmotility, anticonstipation agents, bronchodilators, anti‐epileptics, spasmolytics, sleep medications, and behaviour medications such as stimulants, antipsychotics or anxiolytics (Hogg, 1992; Kapell et al., 1998; Zijlstra & Vlaskamp, 2005). Mothers of children with severe and profound intellectual disabilities experience many challenges in giving medicines: difficulties in physical administration, manipulation of medication, covert administration and alternative formulations (Doyle, 2020a).

Although longer life expectancy and the advent of improved home‐based care is a positive development for children and their families, it creates specific demands on the whole family. When delivering home‐based care, parents repeatedly take on the challenge of managing their child's care hour to hour, often undertaking healthcare interventions such as medication giving and assessing clinical status (Doyle, 2020a, 2020b; Toly et al., 2012). The aim of this study was to explore mothers' lived experience of giving medicines to children with severe and profound intellectual disabilities. Objectives included identifying the range of activities mothers giving medicines undertake and any other pertinent issues.

## 2 METHODS

The study adopted a hermeneutic phenomenological approach using multiple face‐to‐face interviews and participant diaries for data collection. Thematic analysis was guided by the work of van Manen (1990). The study was ethically approved by both the University and service providers through completion of an ethical application and review by an ethics committee.

### 2.1 Sample and recruitment

Purposive sampling was used to identify potential participants to include mothers of children with severe and profound intellectual disabilities who experienced the phenomenon of giving medicines. As mothers are more likely to be the direct caregiver, fathers were not invited to partake in this study as mothers were deemed more likely to have experience of the phenomena. Inclusion criteria required the child they were referring to was aged, between 2 and 18 years of age who needed medication administered daily. The sample was accessed through six service providers who deliver services to children with severe and profound intellectual disabilities in the Republic of Ireland. Recruitment began following receipt of ethical approval. Each service appointed a gatekeeper who distributed information packs to potential participants who fulfilled the inclusion criteria. A total of 46 information packs were distributed, generating 17 responses with final recruitment of 15 participants.

### 2.2 Procedure

Multiple data sources can be used in hermeneutic phenomenology, and in this study, face‐to‐face interviews and participant diaries were adopted over a 17‐month period. Any participant who expressed an interest in the study was contacted by phone to discuss what was required of them before agreeing to participate. Informed consent was received by the researcher prior to the audio recorded, unstructured, first face‐to‐face interviews. All participants were invited to take part in further interviews. Some participants agreed to be interviewed second and third times, using a semistructured approach allowing for in‐depth exploration of the phenomenon. This permitted an in‐depth review of what was previously said and further clarification on key issues. As an adjunct to interviewing, all participants were also asked to keep an account of happenings in relation to giving medicines in a diary given to them at first interview, complementing the interview findings. The diary allowed for collection of data that would enrich and confirm the data already collected during interviews and to clarify and seek responses to questions inadequately explored during interviews. The resulting rich data were used to explore participant situations in the interview process and to confirm the findings in subsequent interviews. Diaries were unstructured but used prompts in the instructions to encourage each participant to document their experiences that were significant to them. Mothers were reminded that there was no right or wrong way of documenting their experiences. Of the completed diaries, some had multiple diary entries, and some had minimal diary entries. A total of 28 interviews and 7 diaries were completed.

### 2.3 Data analysis

Thematic analysis of both interviews and diaries was aided by guidelines to approaching hermeneutic phenomenology, the work of van Manen (1990) who advocates using three methods to isolate thematic statements that attribute meaning to a phenomenon. The process of thematic analysis (Table 1) began at the first interview, through return of completed diaries, and continued to completion of the write‐up of findings. The selective reading approach was assumed involving thorough scrutiny of the text, with the aim of revealing statements or phrases that seem essential to the experience being described (van Manen, 1990). Adding depth to the analysis, the holistic reading approach was also adopted where the text was viewed as a whole and notable phrases that captured the fundamental meaning were identified. This was further presented in line with the five existential themes that are fundamental to the lifeworld of all human beings according to van Manen (2014): ‘lived space’, ‘lived time’, ‘lived body’, ‘lived human relation’ and ‘lived things’. The existential of ‘lived time’ is applied in presenting the findings outlined below. Data were managed using NVivo 11 qualitative data analysis software. Pseudonyms are adopted throughout, and labelling is adopted in the presentation of quotes: Int1, Int2 and Int3 indicate which interview the quote refers to, whereas D refers to diary entries.

**TABLE 1:** Process of data analysis

Van Manen's (1990) guidelines	Phase of data analysis
*Turning to the nature of lived experience*	Transcribing, reading and rereading
*Turning to the nature of lived experience*	Constructing a qualitative database
*Investigating experience as we live it*	Selective reading approach—creating initial themes
*Reflecting on the essential themes which characterize the phenomenon*	Holistic reading approach—creating essential themes
*Describing the phenomenon in the art of writing and re‐writing* *Balancing the research context by considering the parts and whole*	Writing
*Describing the phenomenon in the art of writing and re‐writing*	Adopting an existential approach

## 3 RESULTS

**FIGURE 1:**
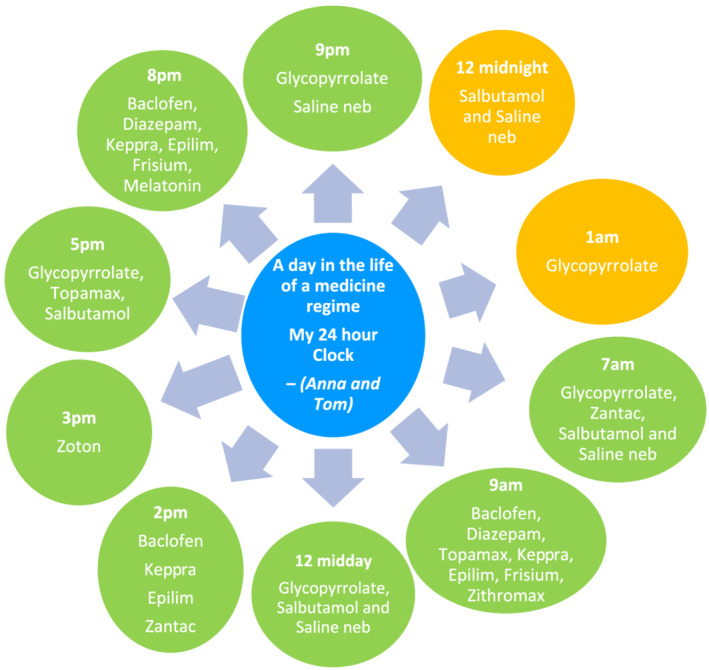
A depiction of the medication giving in a typical daily routine for Anna and Tom. In this case, Tom was prescribed with 17 medications, requiring 13 daily medications and 4 PRN medications depending on his health status, resulting in a total of 33 drug administrations per day. This example is reflective of other mothers' daily medication giving pattern also (yellow signifies those medications given in the night‐time, whereas green signifies the medications given during daytime hours)

**FIGURE 2:**
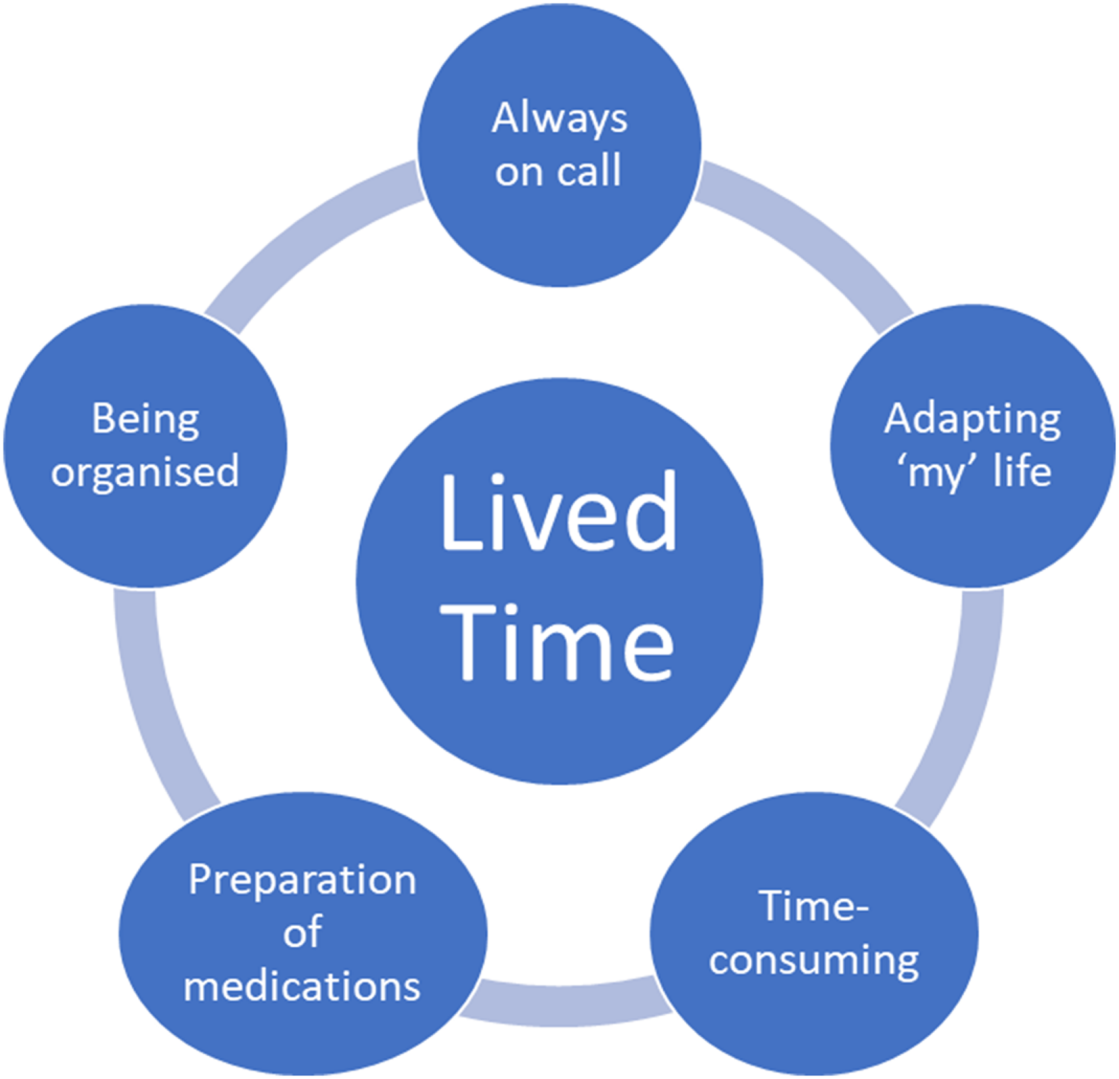
Lived time—themes

The existential dimension of ‘lived time’ provided an understanding of mothers' daily lives in terms of their experience of giving medicines to their child with severe and profound intellectual disabilities. All mothers were conscious of time and the demands placed upon their day with many clock watching and some being all consumed by time (Figure 1). Five themes emerged from ‘lived time’: always on call, adapting my life, time‐consuming, preparation of medications and being organized (Figure 2). A summary table of participant children is provided in Table 2.

**TABLE 2:** Summary of participant children

Child name	Age	Degree of disability	Number of medicines prescribed	Route of administration
Aaron	13	Profound	9	PEG Buccal Nebulized
Ava	17	Severe to profound	16	Oral Buccal Rectal Nebulized
Marie	4	Profound	11	Oral Buccal Nebulized PEG
Barry	14	Profound	8	Buccal PEG
Robbie	13	Severe	9	Oral Injection
Eoin	10	Profound	11	PEGJ Rectal
Jake	10	Profound	17	Nebulized Injection PEG
Tom	5	Profound	17	JEJ Rectal Buccal Nebulized
Max	16	Severe	6	Oral Injection
John	17	Profound	6	Oral Buccal Nebulized
Sophie	9	Severe	5	Oral Injection
Todd	11	Profound	6	Oral Buccal
Shay	17	Severe	6	Oral
Emma	14	Profound	13	PEG Absorption
Niamh	2	Severe	7	Oral Rectal PEG Nebulized

### 3.1 ‘Always on call’

All mothers emphasized that the pattern of their daily lives meant that they were always ‘on call’ and carried their mobile phones if away from their child. Whether it was a weekend or holiday time, mothers had to get up and administer medications as ‘they were going by the clock’ (Pam, mother of John—D) and seldom experienced a lie‐in. If a child had epilepsy, any deviation in the time when medications should be given could have led to an increase in seizure activity.

I think sometimes you'd love to just get into bed, especially when you are tired that you just want to sleep and wake up naturally. But you have to actually go by the clock … some morning I would just love to be able to sleep. (Pam, mother of John—Int2)

Mothers described how they planned their lives around knowing they needed to be home if on a night out and up early in the morning to give medications and so restrained themselves when out. Furthermore, mothers acknowledged the constant pace of their caring role and how they were always on alert due to the complexity of care their child required and the necessity of medication giving. The lack of escape from caring as there was no one else to take over was a recurring matter. One mother admitted that respite offered a break from the monotony of medication giving each morning and from being constantly on call:

it's great having a break from the 7am routine because everything is by the clock, even with the other children. So when Eoin is in respite we'd tend to go off for the day and do something totally random with the other kids, which is good. (Grace, mother of Eoin—Int2)

### 3.2 Adapting ‘my’ life

Mothers acknowledged that they adapt what they do daily to suit their child and the routine of giving medicines. One mother described giving medicines as something they needed to do:

it's probably one of those things that you are doing and you just take it for granted. It's one of those sort of hidden kind of things that just becomes part of your routine, the bottom line is we do whatever we need to do …. (Siobhan, mother of Niamh—Int1)

Some mothers indicated that they plan their lives with a lot of thought and very little spontaneity exists due to the full‐time nature of their role. One mother talked about going home to visit her parents with her child and family and after having a lovely day catching up, it being undone by the necessity to ‘get in’ (Pam, mother of John—Int1) medications into her child who was tired and due to decreased muscle tone found it difficult to swallow. Moreover, this mother recognized that she does not go out at night until she has her child in bed and medications given, to alleviate her anxiety and reassure herself he is okay. Another mother admitted that as the child needed liquidized foods for giving medicines, it determined their lifestyle as they could not eat out because of this. Due to the complexity of some medication giving, choices about where mothers could go with their child were limited and compromise was required:

Life is just compromised from day one, and it's just either (husband) is here or I'm here. Or if we have to go somewhere we'll work it round it that we'll give the meds, or we'll do it after or before … But really, giving the meds anywhere else but here or in your mother's house, or in a house … You know, you could not do it in a shopping centre. It's just not happening. There's too many variables with this dissolving and putting it in cups and drawing it up, and you need a counter space, a surface. (Lynn, mother of Emma—Int1)

The need for privacy when administering some medications due to the nature of the route of administration such as rectal was also highlighted and added another layer of complexity.

### 3.3 Time‐consuming

This theme refers to the amount of time that it takes mothers to manage the task of giving medicines and how time and the clock can impact on their daily life. One mother described how time‐consuming it can be giving the medicines to her son, ensuring he gets them. This was further compounded if additional medicines were prescribed such as an antibiotic.

it is so time‐consuming getting them into him and just allowing time. There is not a thing you can do. It's just he's so slow to eat and he's so slow to swallow that it's not like just, ‘Here's your tablets, take your tablets’, that's it. He cannot just swallow them. You just have to allow the time for it … So you could be talking about the antibiotics, you are talking at least probably twenty minutes before we'd get it into him … We dread to hear he's on the antibiotics. (Pam, mother of John—Int1)

The challenge of not being able to just give medicines using a spoon and the resultant difficulty in swallowing for the child was highlighted, further lengthening the time it took to give medicines. Mothers also talked of family and friends not understanding the time constraints placed on their daily lives due to giving medicines. Morning time was a particularly pressurized period in the day.

I get Aaron up in the mornings, first thing I do, straight away, is his meds, start to feed, and then I'm on to the nebulisers. So he's on his nebulisers and I'm flying. Like there's three nebulisers he has to get in the morning. So it does take a while … Hard. I'm finding it, especially on a school morning, trying to get the meds into him before he goes to school. (Catherine, mother of Aaron—Int1)

Time taken to prepare the medicines and associated equipment for respite took quite a while. Additionally, time was also spent ensuring you had enough stock of medicines and if not you were on the phone ordering either the prescription from the GP or the medications from the pharmacist, completing an informal inventory of your supplies or collecting medicines from the pharmacy. Mothers who had to administer multiple medications on any given day cited the reality that everything else got left undone. ‘You're against the clock the whole time’ (Olivia, mother of Marie—Int1). One mother described her day, trying to get medicines into her child being very time‐consuming especially when there were other children in the mix.

we crush all her tablets and we are trying to get the others ready at the same time and try and eat a bit yourself … We did consider splitting Marie's meds up four times a day, so that we had smaller amounts to give her. But it's not practical … to sit with Marie four times a day for meds. Then you have to get them into her and … try and get her ready and get dressed and ready for school. (Olivia, mother of Marie—Int1)

This mother also conversed about how deterioration in her daughter's health meant an increase in medicines and resulted in more time being consumed by medicines management. She also reflected on Christmas time and how her husband was at home more and to give her a break undertook the medicines management, making her appreciate how long the process actually takes. Similarly, one mother expressed how she was so used to giving medicines; this study made her reflect on it. The time required for preparing and giving medicines was a real concern especially for those mothers who had several medicines to administer:

for 24 hours, there's a medicine for every hour, and I'd say I probably spend about two hours a day getting medicines done up … Because for each medicine you give him, there's a pre and post flush …. (Anna, mother of Tom—Int1)

### 3.4 Preparation of medications

All mothers discussed the preparation of medicines and what this meant for their daily lives. This study made them think about the preparation process and realize what it requires of them. With other children in the house, school runs and in some cases work commitments, being prepared was a top priority and a time‐saving strategy also. In the preparation process, mothers acknowledged ‘drawing them all up at night’ (Liz, mother of Jake—Int2), whereas others did not do this but prepared the daily medications in the morning. One mother described her level of organization the night before, and it is notable how complex this becomes when a gastrostomy tube is being used as the route of administration:

… the ones that I can get ready, I do. Drawing up the meds and leaving the ones that are needing to be dissolved in their little cup, and the ones that need to be crushed—crushing them and getting the right volume of fluid for them. I draw all that up and all the flushes up. I draw about 40 syringes up the night before I have everything literally ready to go because the morning is very busy and he gets a bus to school and I've to get twelve meds into him before that …. (Liz, mother of Jake—Int2)

Furthermore, preparation was influenced by the medicines prescribed with some requiring preparation just before administration, meaning you could not be organized in advance. Some mothers described the technical elements of preparing medicines, and this resulted in a more difficult administration process, especially if reconstitution and manipulation of medication were required. Mothers identified their own way of giving medicines. This was interesting as no two mothers had the same way of administering but clearly adapted to their child's individual needs.

I crush the Vitamin D tablet, the Kepra tablet. I've already put the 30ml of peppermint water and yoghurt, honey and her 5ml of Potassium Citrate into the bowl. I thicken them up and then I do not add the Vitamin D and the Kepra until just before I feed her … I just have this idea that I do not want the Kepra interacting with other stuff for the 20 minutes it takes me to get her up. So I have it mostly ready and thickened …. (Gillian, mother of Ava—Int1)

As medicine giving requires a degree of accuracy to avoid any risks to the child, mothers discussed the precision adopted in preparing the medicines for administration. Mothers assumed procedures for ensuring safety, an important element especially when tablets or liquids looked similar in shape, size or colour.

I take all of the bottles out of the medicine cupboard and line them up, but when I've drawn the first one I close the bottle, put it back in the box and put it in the cupboard, so that there's no risk that I'll get confused about what I did or did not already draw into syringes. (Gillian, mother of Ava—Int3)

A level of precision was also required in terms of measuring the various medicines especially if they required reconstitution, crushing or dispersal and the child was not on a full dose, which meant mothers were measuring portions of medicines and discarding the remainder. Additionally, flushes for gastrostomy tubes had to be considered. This level of precision was also used in terms of ensuring safety was a top priority with one mother acknowledging she cuts the tablet packs into groups of 3 so she knows she only needs to take out one piece of the packet per administration, and in turn, she felt this was safer than having the full pack out on display. Precision was also made easier when mothers adopted strategies such as using a specific size syringe for drawing up medicines or for administration, for example, 5‐ml syringe for 5‐ml medication. One mother specified that many of the medicines her child was prescribed were only available in tablet form and required crushing and then dissolving further in liquid in order to administer more easily, but this took more preparation time. Being precise was also described by one mother relating to preparing an injection for her child. She recognized her commitment to ensuring there was no scope for error by using the correct size syringe for the dose that was required:

I transfer his Anakinra to a syringe. I transfer from, it's 100 mg in 0.67 ml … it's a bit tricky, and you have to transfer that into an insulated syringe. He gets 0.47 ml at the moment and I give that in a 0.5 syringe … I really am a stickler … I try to use the correct size syringe, so like, he's on 0.8 of Sildenafil, so I use a 1ml syringe. He's on 2ml of Motilium, so I use a 2.5ml syringe. It just leaves less room for mistakes. (Liz, mother of Jake—Int1)

Precision was also adopted by mothers in terms of timing and how it was important that their child be positioned correctly, often upright in a chair, to support the ingestion of medicines. This became more problematic when it was night‐time and the child had to be removed from bed to ‘give medicines’.

### 3.5 Being organized

This theme refers to the level of organization required in daily life relating to giving medicines and encompasses concerns about ensuring medications are administered at appropriate times without delays, being organized in terms of travelling and ensuring medications are packed. Organization is required to ensure medications are administered on time especially in the case of AEDs.

… Because he's on the anti‐convulsant medication, it's just imperative we get the medication into him. We cannot go anywhere without being so organised with taking everything. I'm from the country, and if we go home I have to always make sure I have it all with me. (Pam, mother of John—Int1)

All mothers indicated the level of daily organization required especially in the morning time and also throughout the day.

Jake is on 17 meds daily and he's on them numerous times a day … in the morning he's on 12 medications and a Flixotide inhaler as well, or a nebuliser if he's unwell … So I draw up all my meds the night before. He goes to school at quarter to nine in the morning, so I need him to have his meds before he's going on the bus. Then I have his meds drawn up for when he comes home from school. Then at nine o'clock he gets 8 meds. Then at ten o'clock he goes onto his water … You just have to be ultra organised …. (Liz, mother of Jake—Int1)

This mother also admitted that with the requirement to be so organized that sometimes she felt it was the only thing she could keep on top of. Additionally, mothers also relayed the importance of ensuring being organized included having the medications ordered. Interestingly, one mother described her checking process as akin to preparing a ‘shopping list’ (Lynn, mother of Emma—Int1). Mothers also indicated that ensuring you kept copies of prescriptions was important especially when several people were involved in the process and to ensure the child got the correct drug and dose.

you really have to micro‐manage and make sure that if the hospital is faxing the GP, they post the original to me so that I can make sure that what I'm getting from the GP is what the hospital prescribed. (Gillian, mother of Ava—Int3)

## 4 DISCUSSION

The findings provide an understanding of mothers' daily lives in terms of their experience of giving medicines to their child with severe and profound intellectual disabilities. The concept of time and the impact of giving medicines were apparent, mothers being ‘always on call’ and the constant full‐time pace of their caring role evident. This is to be expected, largely due to the nature of disability and the associated medical conditions requiring medication management (Brenner et al., 2018, 2020; Doyle, 2020a; Green, 2007; Nakken & Vlaskamp, 2007). Often, these conditions are complex and require a demanding schedule of care (Brenner et al., 2018; Davies & Carter, 2013; Davis et al., 2014; Luzi et al., 2019; Nakken & Vlaskamp, 2007; Toly et al., 2012). Furthermore, nurturing a child with severe and profound intellectual disabilities develops into a lifelong responsibility placing enormous burden on families (Carter & Bray, 2017; Coad et al., 2014). This was evident here with mothers afraid to deviate from specific times for giving medicines due to the risk of deterioration in their child's condition, resulting in mothers adhering to a 24‐h schedule of care.

The difficulty highlighted by mothers surrounds the delivery of relentless complex care within the home setting, something that may historically have been delivered in the healthcare setting, where at least staff got a break (Leiter et al., 2004; Murphy et al., 2021). The intensity and unpredictability of mothers' caring responsibilities as well as lack of escape from their role was evident and is supported within the literature (Bourke‐Taylor et al., 2010; Heaton et al., 2005; Swallow et al., 2011). More specifically, the task of giving medicines was acknowledged by these mothers as an onerous one eating into their caring activities. Similar to this study, the high time demands on parents were explored by McCann et al. (2012, 2015) who identified that parents of children with complex needs carry significant caregiving burden often increasing as the child gets older. However, this particular study emphasizes the task of giving medicines and all of the associated work that is involved in ensuring these are administered on a daily basis often multiple times per day. Furthermore, it was established that the concept of ‘vigilance’ was a large component of caregiving for these parents. This vigilance referred to by Carter and Bray (2017) was also explained by mothers indicating that even if it was a weekend, they still had to get up and ‘give medicines’. Additionally, this study has increased insight into vigilance in the context of monitoring for medicine effect and side effect, all demanding a portion of time for mothers.

The time it took to ‘give medicines’ to children was sometimes considerable within this study. This task is multifaceted and encompasses many elements (Dunworth‐Fitzgerald & Sweeney, 2013) with the child's capacity to take medicines at the forefront of the preparation process. Comparable with the study by McCann et al. (2012), giving medicines was particularly time‐consuming and complex if the child was on multiple medications, required coaxing to take medicines or required small amounts to allow ease of swallowing. The effort endured in giving medicines to children with severe and profound intellectual disabilities is not considered in the literature. However, caregiver burden experienced with this cohort is contemplated and the unpredictability of physical tasks, which may include giving medicines are alluded to (Edelstein et al., 2017; Heaton et al., 2005; McCann et al., 2015; Nicholl & Begley, 2012; Page et al., 2020). This unpredictability was something mothers experienced and noticed especially when other demands were placed on them.

It is also notable that many of the mothers used several routes of administration for medicines, requiring a diverse array of preparation skills. It became more time‐consuming when a gastrostomy tube was being used as this required a more complex medicalized procedure or when a child's condition deteriorated and determined the need for yet more medications. Using a gastrostomy tube for giving medicines requires alteration to drug formulation, and not all medicines are available in liquid form, the recommended formulation for administration using a gastrostomy (Wright, 2011). Therefore, alternative approaches to manage tablets are required to ensure the correct consistency to minimize blockage while also maintaining accuracy and precision (Wright & Kelly, 2012). Additionally, consideration of the time required for maintaining a continuous supply of medicines, having up‐to‐date prescriptions and paperwork and linking with the GP and pharmacist to ensure there are no delays in securing the medicines is not pondered in the literature. Mothers' desire for additional support from healthcare professionals including the GP and pharmacist was evident.

Mothers acknowledged that they adapted their daily life to suit their child and the routine of giving medicines, with few spontaneous activities or social outings planned. Heaton et al. (2005) recognize this need to adapt, also supported by Crowe and Michael (2011) with an acknowledgement that typical days do not really exist for mothers of children with severe and profound intellectual disabilities. Finding the time to do anything other than care is difficult and stressful with the literature identifying specific difficulties for mothers in terms of employment, leisure activities or social interaction (Brandon, 2007; Carnevale et al., 2008; Silibello et al., 2016). Although some mothers in this study worked outside the home, this required immense planning and organization for their child with severe and profound intellectual disabilities. Diehl et al. (1991) and Woodgate et al. (2015) suggest that one parent usually ends up taking on the full‐time caring role. Evidence also indicates that the daily burden of routine and continual responsibilities can result in physical and emotional over burden for carers (Woodgate et al., 2015) with having enough time at the core of their experience. In discussing the dimension of ‘lived time’, it is clear that mothers' experience of giving medicines is very much relentless, requiring continuous input especially when there is a demanding schedule of challenging and complex medicine administrations.

### 4.1 Implications for clinical practice

Although caregiver burden is addressed in the literature, the intricacies of giving medicines are less so. There are implications for various other cohorts of children with continuing medication requirements: those with complex healthcare needs and those with chronic illness. There appears to be no standard system of practice in Ireland for supporting mothers of children with severe and profound intellectual disabilities in giving medicines. Mothers admitted not being asked about their experiences and therefore placed value on participating in this study, providing a rare opportunity to discuss their daily lives. It is important that health professionals develop an understanding of the task of giving medicines and the associated time required to manage these medicines on a daily basis, appreciating the challenges experienced by mothers. Formal education for mothers (parents) needs to be developed to encompass all elements related to giving medicines, in turn empowering them to be confident and competent in this task. The importance of supportive relationships from professionals especially the general practitioner, pharmacist and hospital is also key in providing supportive elements to mothers' daily role. With the volume of medications and administrations, development of a single medication administration record for use at home is welcome. This should form part of the medication passport where a record of daily administration is recorded. The availability of respite care is also recommended as a positive coping strategy for mothers to have a break from the daily task of giving medicines.

### 4.2 Limitations

Published research on this topic area has been limited; thus, this study offers a first‐hand understanding of mothers' lived experience of giving medicines to children with severe and profound intellectual disabilities. Although this study offers insight into a topic area often forgotten by professionals and not prioritized, it is worth noting that the purposive sample engaged in this study may have chosen to partake as they had experienced specific difficulties in giving medicines. Furthermore, the experiences captured are a moment in time, and subsequent experiences might sway mothers' understanding of the phenomenon. Additionally, fathers who might be direct caregivers were not included in this study.

## 5 CONCLUSIONS

The findings of this hermeneutic phenomenological study have enabled exploration and familiarity with the lifeworld of mothers of children with severe and profound intellectual disabilities. It is clear that mothers are ‘always on call’, rarely experiencing spontaneity in their lives as they are stifled by the routine of giving medicines. Time spent in giving medicines determined how they live by the clock. The necessity to be prepared and organized was highlighted as important in ensuring children got their medications on time and safely. This study offers meaning on the phenomena of giving medicines. It was concluded that this experience is a relentless and challenging one, yet appears invisible as an element of care in professional discourse. This study addresses a gap in understanding of how mothers experience giving medicines and recognizes the family's central role in caring for a child with severe and profound intellectual disabilities. It may be useful in facilitating the development of care especially at a time when the numbers of children with severe and profound intellectual disability are increasing. The findings will also contribute to enhancing the support and care of mothers and children with severe and profound intellectual disabilities and provide a platform for further deliberation.

## CONFLICT OF INTEREST

The author reports no conflicts of interest.

## Data Availability

The data that support the findings of this study are available on request from the corresponding author. The data are not publicly available due to privacy or ethical restrictions.
